# Determinants of immigration strategies in male crested macaques (*Macaca nigra*)

**DOI:** 10.1038/srep32028

**Published:** 2016-08-18

**Authors:** Pascal R. Marty, Keith Hodges, Muhammad Agil, Antje Engelhardt

**Affiliations:** 1 Department of Population Health and Reproduction, University of California Davis, USA; 2 Junior Research Group Primate Sexual Selection, German Primate Center, Göttingen, Germany; 3 Reproductive Biology Unit, German Primate Center, Göttingen, Germany; 4 Faculty of Veterinary Medicine, Bogor Agriculture University, Indonesia; 5 Faculty of Science, School of Natural Sciences and Psychology, Liverpool John Moores University, UK

## Abstract

Immigration into a new group can produce substantial costs due to resistance from residents, but also reproductive benefits. Whether or not individuals base their immigration strategy on prospective cost-benefit ratios remains unknown. We investigated individual immigration decisions in crested macaques, a primate species with a high reproductive skew in favour of high-ranking males. We found two different strategies. Males who achieved low rank in the new group usually immigrated after another male had immigrated within the previous 25 days and achieved high rank. They never got injured but also had low prospective reproductive success. We assume that these males benefitted from immigrating into a destabilized male hierarchy. Males who achieved high rank in the new group usually immigrated independent of previous immigrations. They recieved injuries more frequently and therefore bore immigration costs. They, however, also had higher reproductive success prospects. We conclude that male crested macaques base their immigration strategy on relative fighting ability and thus potential rank in the new group i.e. potential reproductive benefits, as well as potential costs of injury.

In gregarious species, dispersal of individuals evolved as a consequence of selective pressures from within group competition and inbreeding avoidance^1^,^2^,^3^. Dispersal is a crucial event for the migrating individual with major implications for its future survival and lifetime reproductive success^4^. In addition to potential reproductive benefits of dispersal, there are, however, also potential costs^5^,^6^, both during the transition period (i.e. between departure from the initial group and subsequent immigration into the new ones), as well as directly during the immigration process itself^4^,^5^,^6^,^7^,^8^,^9^,^10^,^11^. During the transition period, individuals transferring from one group or territory into another, face increased predation-risk and restricted access to known food resources^4^,^12^. For group living animals, resistance from resident individuals, often leads to fights resulting in severe injuries, and represents a significant cost to the immigrating individual^10^,^11^,^13^. If payoffs for specific behaviours such as dispersal hinge on internal states, the evolution of condition-dependent plasticity is likely^14^,^15^. Accordingly, potentially migrating individuals have a specific set of preconditions, such as fighting ability, which are likely to directly influence the decision whether to migrate or not. If costs are too high, individuals are not expected to migrate, whereas migration is expected when benefits outweigh costs. Thus there should be a selective force on individuals to align migration strategies to their specific condition and to develop strategies that increase their potential reproductive benefits and/or lower the costs of dispersal.

Within mammals, primates are probably the best studied taxa regarding dispersal and migration behaviour. As in most other mammalian taxa, males are usually the main dispersing sex, with emigration out of the natal group typically occurring around the time of puberty^16^. In contrast to most other mammals^17^, however, adult male primates are known to migrate and join new groups several times during their lifespan^16^,^18^. In view of the high costs of migration, it is to be expected that males have evolved strategies to reduce these costs, and such strategies have now been described in a variety of primate species. These strategies have been explained by potentially reducing immigration costs. For instance, by immigrating into neighbouring groups and thus reducing the time spent alone, the costs of the transition period can be reduced considerably. Alternatively, transition costs can potentially be eliminated by immigrating during intergroup encounters^10^,^19^,^20^,^21^,^22^,^23^,^24^. In some primate species, males have also been observed to potentially reduce immigration costs posed by the resistance of resident males by immigrating with peers and kin, or into groups in which previously immigrated peers or kin are present^10^,^19^,^25^,^26^. In addition, by acquiring social knowledge of the target groups resident males prior to immigration (e.g. during intergroup encounters or previous observations of neighbouring groups during roaming), males may be able to assess the potential costs and benefits of immigration in a new group and base transfer decisions on the acquired knowledge^9^,^22^,^27^. Further, an increased number of immigrations during male hierarchy instability caused by high-ranking immigrants^22^,^28^,^29^,^30^ has been observed^22^,^31^. In this situation, subsequently immigrating males may experience reduced costs, since male-male cooperation against intruders may be reduced; and within group male-male aggression increased^29^ when resident males have to consolidate their position within the group. Evidence for such a scenario derives from vervet monkeys (*Chlorocebus aethiops sabeus*)^32^.

Given the number and nature of potential costs of dispersal, migrating males can be expected to be under high selective pressures to optimally time and locate immigration. For primates, it has been hypothesized that immigration decisions are based to varying degrees on the male cohort and the number of adult females of the new group as a function of a species’ degree of male-male competition and reproductive skew^33^. In species in which male mating competition is mostly scrambled and male reproductive skew low, male rank is of relatively little and the number of females in a group of relatively high importance for a male’s individual reproductive success. Dispersing males of such species are therefore supposed to base their decision on a group’s sex ratio rather than on the characteristics of group males^33^. For species in which male-male competition within the group is frequent and paternity is highly concentrated to top ranking males, in contrast, it has been suggested that males mainly base migration decisions on the characteristics of resident males and the chances of achieving a high rank within a short period of time. In-between these extremes, the degree to which males should base their decision on either of the two parameters is supposed to depend on the degree of reproductive skew and thus on the potential reproductive benefit a male would achieve either through trying to gain a high rank or through living with an increased number of females.

The degree of male-male competition and reproductive skew can also be expected to play an important role for strategies to reduce migration costs. Whereas in species with low degree of male-male competition and reproductive skew, resistance of resident males against immigrating males can be expected to be low, the opposite should be the case in species where males fight fiercely over access to females and where reproductive skew is high. Accordingly, species with low reproductive skew show low male-male competition, escalated fights between males are rare, immigration costs comparably low and new immigrants often start at the bottom of the hierarchy (e.g. Japanese macaques, *Macaca fuscata*^34^). In contrast, in species with high reproductive skew, escalated fights are common and costs of immigration in terms of injuries high (e.g. Hanuman langurs, *Semnopithecus entellus*^33^,^35^). Particularly in these species, males can be expected to develop strategies that potentially reduce immigration costs.

In addition to inter-specific differences in migration decisions, there may also be inter-individual differences within the same species since not all males may face the same costs and benefits when dispersing. Inter-individual differences in migration decisions can particularly be expected in species with a high male reproductive skew, i.e. in species in which variation in fighting ability leads to significant differences in reproductive benefits. Similar to the species specific predictions for migration decisions, males can be expected to base selection of a new group on the male and female cohort of the potential target group^33^. Again, males with a high fighting ability are supposed to first and foremost choose groups in which they can quickly achieve high rank, i.e. where resident males are comparatively weak. An example for this are long-tailed macaques where challenges of the alpha male are more successful the longer the alpha male held tenure, i.e. the older alpha males were and the more likely they had passed their prime^33^. Males with low fighting ability, in contrast, are supposed to particularly immigrate into groups with an absolute and/or relative higher number of females since this variable is more important for their reproductive success^33^. At the same time, these males who can only expect a low reproductive benefit from migration can be expected to reduce the costs of immigration as much as possible. This could, for example, be achieved by migrating with other males who can serve as allies against resident males^26^. Alternatively, these males could align migration to that of males with high fighting ability, which usually cause group instabilities^28^,^29^,^30^ that may distract from immigrating males with low fighting ability. Hence, while males with high fighting ability should be concerned with timing their immigration to a period when success of reaching a high rank is most likely, those with low fighting ability should rather align their time of immigration to that of others, e.g. that of strong males.

Although several studies on primates have investigated male migration decisions and strategies, to our knowledge only one so far has looked at intra-individual differences. Interestingly, data of this study, conducted on chacma baboons (*Papio hamadryas ursinus*), a species with high male reproductive skew, did not support the above mentioned relationship between reproductive skew and immigration decision^36^. Opposite to what actually has been predicted^33^, high quality male chacma baboons chose new groups based on the number of females whereas for males of low quality, neither absolute nor relative number of females in the group seemed to play a role. All other studies on primate male migration decisions so far either concerned species specific strategies or compared differences between species^9^,^19^,^22^,^28^,^33^,^37^,^38^. Thus there is clearly a lack of studies focusing on inter-individual differences in migration decisions.

The aim of our study therefore was to investigate inter-individual differences in immigration decisions based on expected costs and benefits of immigration in a species in which inter-individual differences can be expected to be high. Crested macaques (*Macaca nigra*) are such a species. They live in multi-male multi-female groups in which the top three males of the hierarchy monopolize the vast majority of paternities (93%) (Engelhardt *et al*. under revision). Males fight fiercely for high-rank and alpha male tenures are exceptionally short^39^. Migration from one group into another is very common and males are known to migrate several times in their life. In this species, inter-individual differences in fighting ability can thus lead to high inter-individual differences in reproductive output and males should align their migration strategies accordingly. Crested macaques are moderately seasonal according to the definition of van Schaik^40^ whereby females can give birth year round. Male-male competition can therefore be expected to vary only moderately throughout the year. Given the high reproductive skew in favour of high-ranking males, we predict males who achieve high rank after immigration (i.e. reaching rank 1–3 in the new group; high rank achievers) to be males with high fighting ability who base migration decisions on characteristics of the target group’s male cohort, i.e. to select groups i) with a low number of resident males and ii) with a potentially weak alpha male due to a long tenure. In addition, we included the number of females potentially predicting male immigration as seen in chacma baboons^36^. (iii) For males achieving all other ranks (low rank achievers), we predict them to be males with low fighting ability who iv) preferably immigrate into groups with many females. We additionally expect these males to v) show parallel dispersal (disperse together with other males that may serve as potential allies against resident males) and to vi) align immigration to that of high rank achievers/immigrants of high fighting ability, either by immigrating together with such a male or shortly after, in order to reduce immigration costs by exploiting circumstances of male hierarchy instability. Additionally, we investigated whether immigrating males differed in benefits in terms of group tenure depending on whether they achieved a high or low rank in the new group.

## Results

### Immigration time pattern

**Figure 1:**
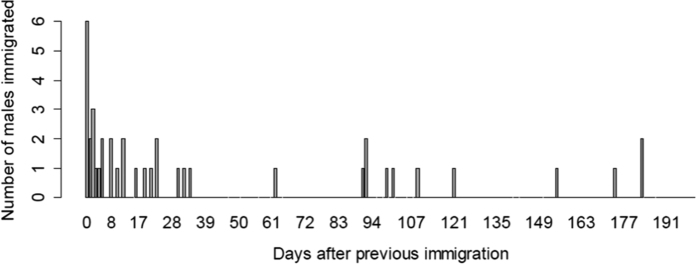
Time distance (in days) between two immigrations and the number of males immigrating into the same group.

Immigration of a new male into a group occurred on average every 81 days (range 16–182 days). A change-point analysis revealed that immigrations occurred non-randomly with 74% of all males immigrating within 25 days of another male (before or after) (Fig. 1).

We found 15 different immigration events where multiple males immigrated into the same group within 25 days of each other. In two out of the 15 immigration events we could observe males immigrating from the same group of origin whereby in only one case we saw parallel dispersal with two males from the same group of origin immigrating together at the same day. In another six events, parallel dispersal could be excluded due to the known migration history of the males. Within each immigration event, the first immigrant was always a high rank achiever (reaching one of the top three ranks).

### Inter-individual differences in immigration patterns and rank acquisition

From the observed 57 immigrations into one of the four study groups, we could determine a David score for 40 males. Out of these, 24 (60%) reached one of the top three ranks in the new group (high rank achievers), and 16 (40%) reached a rank below these positions (classified as low rank achievers).

In terms of timing, 22 (55%) males immigrated independent of other immigrations (with no immigrations into the same group in the previous 25 days; further called pioneers), while 18 (45%) followed another male into the group within 25 days (further called followers). High rank achievers immigrated statistically significantly more often as pioneers (N = 18, 75%) than did low rank achievers (N = 4, 25%) (Chi-square test, χ^2^ = 7,782, P = 0.005).

In all 18 cases, followers followed a high rank achiever, and no male ever followed a pioneer who did not achieve a high rank. Followers thus followed statistically significantly more often a high rank achiever than expected randomly (Binomial test; p < 0.0001).

The subsequent tenure length of immigrated males showed a high variation ranging from 2 to 1797 days, but did not statistically significantly differ between high- and low rank achievers (Mann-Whitney U-test, U = 178, N = 36, P = 0.585).

### Impact of group constellation of the target group and potential other groups on the individual immigration decision

Model 1 revealed that high rank achievers immigrated significantly more often into a group with a higher number of males than in the most favourable control group (i.e. the group with the lowest number of males) (P = 0.040, estimate = 1.26, SE = 0.61). Alpha male tenure did not significantly differ between potential groups (Table 1). Model 2, investigating low rank achievers, showed that these males did significantly more often immigrate into a group where the resident alpha male had a short tenure (P = 0.010, estimate = −7.12, SE = 2.78). The number of females in the target group did not statistically significantly differ from the most favourable group (Table 2). After controlling for multiple testing using the Benjamini-Hochberg correction, non-parametric tests revealed the same results for both high and low rank achievers (see supplementary material).

### Male-male competition and immigration costs regarding injuries

Observed direct male-male challenges were brief, and subsequent escalated fights between males were decided within five minutes, often with one male becoming injured. We found high rank achievers to be significantly more likely to become injured in the first three weeks following the immigration than low rank achievers (two tailed Fisher exact test, P =  0.038). 35% (N = 8) of high rank achievers got injured whereas no low rank achievers did.

Eight of twelve (67%) resident alpha males got injured at the time of replacement (whereby one injury was potentially a consequence of an intergroup conflict). Seven of the injured males left the group upon replacement and one stayed in the group. Two other replaced males without injuries also left the group immediately after replacement. In addition, we observed one case in which an alpha male got challenged, won the fight but both males got badly injured. This alpha male lost his position within a couple of hours and dropped severely in rank. Two days later, he left the group.

## Discussion

Our results show that immigrations by male crested macaques typically occur during immigration periods whereby several males immigrate around the same time. During such immigration periods, males follow two distinct strategies depending on potential costs and reproductive benefits of immigration. Males entering the new group at the upper end of the male hierarchy immigrate significantly more often independent of others (pioneers) and trigger an immigration period. In our study, these males experienced significantly more injuries (i.e. costs), but were also those with far higher potential for high reproductive success in the new group due to the strong reproductive skew in favour of high-ranking males found in the study population (Engelhardt *et al.* under revision). In contrast, most males not achieving any of the top three positions in the group (followers) time their immigration to the immigration of pioneers who have achieved high rank. We never observed any costs in terms of injury in any of these low rank achievers. At the same time, they can also only be expected to achieve low benefit in terms of reproductive output in the new group.

Our results can be interpreted in two different ways: first, rank achieved in a group is a direct consequence of the immigration strategy with pioneers generally achieving a higher rank than followers. Alternatively, males are aware of their potential for achieving a high rank and choose the immigration strategy accordingly. The first explanation seems rather unlikely. First of all, would all males immigrating as pioneers achieve high rank (and thus potentially high reproductive success) per se, males would not be expected to ever immigrate as followers. In our study, 45% of males, however, immigrated as followers and not as pioneers. Furthermore, there would be no good explanation for why pioneers, but not followers, would have the opportunity to achieve high rank. Secondly, in a species where high rank is very beneficial for males of high rank and where males therefore fight so fiercely for it, it is very unlikely that just any immigrating male would be able to achieve high rank upon immigration. It thus seems more likely that pioneers are males of high fighting ability that did not make the timing of immigration dependent on the occurrence of other immigrating males. In crested macaques, males may fight fiercely for rank and get heavily injured. In a previous study, we could show that they either achieve a high rank directly upon immigration (e.g. within a couple of minutes) or they avoid conflicts with the resident males and thus end up with low rank^39^. Male-male alliances or coalitions have never been observed in the context of immigration. It therefore seems that male fighting ability is at least an important determinant of future rank in this species.

Our results do not support our prediction that high rank achieving male crested macaques base their immigration decision on characteristics of the target group males (i.e. number of males, alpha male tenure; see also^33^) or females^36^. Males did not immigrate into the group with the lowest number of competitors. In most cases, there was at least one group nearby with a lower number. Similarly, males neither immigrated into groups where the resident alpha male already had a long tenure nor into those with more females. The reason for this phenomenon may be that other characteristics of resident males, for example individual fighting ability, may be more important than their absolute number, particularly in a species where male coalitions are rather rare (Neumann *et al.* in prep.). Similarly, tenure length may not be a good measure of alpha male fighting ability in crested macaques since it can vary tremendously between individuals and males with longer tenure may also represent the stronger ones^39^. Interestingly, a surprisingly high percentage of pioneers achieved the alpha male position upon immigration. This position is often achieved by primary migrating males who remain in their natal group until they reach maximum body size and therefore maximum fighting ability^39^. It thus seems that rather high body mass, i.e. fighting ability than the target group’s constellation is the key factor determining whether males challenge high-ranking males for their position or not. Fighting ability might therefore determine the timing of migration for primary migrating males which often end up being lead immigrants in immigration periods. However, we could not determine the factors influencing the decision in which group to immigrate.

Also contrary to our prediction, males with lower fighting ability did not show a preference for groups with many females. They did, however, preferentially immigrate into groups with a recent changeover in the alpha position (short alpha male tenure). Consequently, immigrations often occurred in clusters and low rank achievers immigrated as followers. This finding is in line with our prediction that particularly low rank achievers in species with high reproductive skew should migrate together to exploit circumstances of group instability. By doing so, they may substantially reduce the costs imposed by resident male resistance. Since follower males in crested macaques appear to be unlikely to receive injuries, the use of group instabilities is likely to represent an adaptive strategy for low rank achievers to reduce the costs of immigration and to facilitate it. Other than predicted by van Noordwijk and van Schaik^33^, however, our results suggest that low rank achieving crested macaque males do not base their immigration decision on prospective reproductive benefits, but on the potential to reduce costs during immigration.

In order to exploit circumstances of group instabilities in other groups, follower males appear to have information not only on the arrival of a new male in a group but also on group instability resulting from immigrations of strong, challenging males. The only way for followers to gain this knowledge in advance is to regularly observe other groups and to wait for suitable immigration opportunities to arise, and this behaviour has been regularly observed for various species^21^,^22^,^41^,^42^. Since crested macaque males often immigrate with previously unknown individuals, the identity of other immigrants does not seem to influence immigration decisions. In contrast, the fighting ability of previously immigrated males seems to be very important since in our study, low rank achieving males who immigrated as pioneers were never followed by other males. In general, the immigration as a follower of low fighting ability seems to be associated with lower costs but also lower reproductive benefits compared to high rank achievers. These reduced costs may be the reason for why group changes by males occur so frequently in crested macaques compared to other primate species.

Even though immigrations are clustered in time, we could not find evidence that male crested macaques preferentially immigrate with peers. Only one out of 28 males with a known group of origin immigrated with a peer. However, 21% immigrated at the same day another male immigrated. Thus, parallel dispersal does not seem to be a typical male strategy in crested macaques. Assuming the case where parallel immigrations occurred was not by chance, some advantages must be conferred for one or both males. Since in crested macaques, males of the same age are likely to be half-brothers due to the high reproductive skew in a group, post dispersal nepotism is likely to increase a male’s direct and indirect reproductive benefit as recently found in long tailed macaques^43^. However, since only a few males immigrated at the same day, and only one pair could be confirmed to show parallel dispersal (and therefore could be potential half-brothers), it is unlikely to be a strategy widely adopted by males. Since male tenure for alpha males is very short^39^, males might not invest time in allies but rather try to get and remain in a high position alone.

To our knowledge, this is the first study investigating individual immigration strategies based on costs and benefits in a mammalian species living in a multi-male, multi-female society. In comparison to other studies focusing mainly on benefits of immigrations, we could show that at least for low rank achieving crested macaque males, migrations are more driven by avoiding costs than by potential benefits. Condition dependant plasticity, as found in crested macaques in this study, can be expected to occur in a variety of other socially living species were individuals have to make migrating decisions. Dispersal behaviour in general remains mostly understudied, and factors promoting dispersal are often of theoretical rather than empirical nature. Therefore, Further studies are needed incorporating alternative migrating strategies when looking at male careers and life history, and ultimately investigating the lifetime reproductive success of males.

## Methods

### Study site and subjects

The study took place in the Tangkoko Reserve in North Sulawesi, Indonesia (1°33′N, 125°10′E) as part of an on-going long term project (Macaca Nigra Project, www.macaca-nigra.org). The reserve ranges from sea level to 1350 m and comprises an area of 8867 ha lowland rainforest^44^. The habitat of the study groups was a mixture of undisturbed primary forest, secondary forest and regenerating former gardens. Wild crested macaques were studied from mid-2006 until the end of 2012 (R1&R2) (Table 3). Two of the observed groups (R1 and R2) have been constantly studied since 2006, whereas one group (PB) has been studied by the project from 2008 onwards. A fourth group (R3) was sporadically followed since 2006, but regular data were only collected from July to December 2012. Group size varied between 60–90 individuals for the three groups R1, R2 and PB whereas the group size for R3 was around 25 individuals. All individuals were fully habituated to the presence of human observers.

Census data on the group males were collected whenever the groups were followed. The three main study groups (R1, R2, PB) were followed on average 4–5 days per week. We therefore could determine the day of entry with an error of 2–3 days. Males were classified as immigrants when they joined a group (also called target group) and were observed to affiliatively interact with resident group members. A changepoint analyses (Change Point Analyzer 2.3; Taylor Enterprises, Inc.) was conducted to reveal significant changes in the mean squared error distribution of male immigrations^45^. 10,000 boot- straps with replacements were run and the confidence interval was set at 99%.

### Behavioural data collection, determination of the dominance hierarchy and fighting ability

Allgroups were regularly observed during all-day follows. During the study period, 57 male immigrations could be observed. Behavioural data on aggressive interactions were collected using focal animal sampling^46^ and *ad libitum* sampling. All data were entered in hand-held computers using spread-sheet software (PTab Spreadsheet v.3.0; Z4Soft). In order to quantify dominance hierarchies, we considered all displacements (approach/leave) and agonistic dyadic dominance interactions between males. All interactions were considered when a clear winner/loser could be determined, i.e. either fights with one animal fleeing, submitting or leaving, threats upon which the threatened individual left, or approach/leave interactions (supplants). For all new immigrants we created winner-looser matrices for agonistic interactions and displacements with all adult males present after the immigration. Depending on the available data, interactions within the first three to six months after the immigration were included in order to obtain an accurate rank. Dominance rank was assessed using corrected normalized David’s score^47^, using the package “Steepness”^48^ in R^49^ based on a matrix of proportions of wins calculated for each dyad. Males whose recorded aggressive interactions were not sufficient to calculate a significant steep and linear David’s score but have been observed to successfully challenge or be challenged by a confirmed alpha male, were included into the analyses. To be able to compare the ranks within and between members of different groups, rank was normalized to range between zero and one with alpha males having the lowest score. Hierarchies were considered unstable for one month after the immigration of a high-rank immigrant as it has been shown for crested macaque groups^31^.

The specific fighting ability of a male was indirectly defined by the rank a male achieved upon immigration into a new group. Males who achieved one of the top three positions in the hierarchy (and therefore are expected to gain reproductive benefits) were considered to be males of high fighting ability. All other males were defined as males of low fighting ability.

### Measurements of injuries as a proxy for immigration costs

Each study animal was checked for injuries (i.e. open wounds, cuts, as well as limping due to potentially broken bones) whenever the group was followed. All observed injuries were recorded.

All measurement as well as all behaviour observation were conducted non-invasively. This research adheres to the legal requirements of the German and Indonesian governments, and adheres to the American Society of Primatologists Principles for the Ethical Treatment of Non-Human Primates.

### Statistical analyses

To investigate the factors triggering male immigration in male crested macaques, we carried out two general linear models (GLM) including data from 26 immigrations where sufficient data on composition and alpha male tenure was available from the group of immigration and from at least one alternative group. One model was conducted for high rank achievers, i.e. males achieving one of the top three position in the new group (model 1), and one model for low rank achievers (model 2). In order to investigate male migration preferences, we compared the condition in the target group to that of other available groups into which the male did not immigrate. These alternative groups were all in close proximity to each other. For each observed immigration, we entered two lines in our data set: one for the condition of the target group on the day of immigration, and one for the most favourable condition in other potential target groups on the same day. For example, in the model for low rank achievers, we compared the actual number of adult females in the target group with that of the alternative group that had the highest number of females. The response variable 0 was the most favourable condition in any of the alternative groups and the response variable 1 was the condition in the target group. In that sense, positive estimates indicate higher values of the predictor in the target group. The predictor variables for high rank achievers were: 1. the effect of the number of males in a group, 2. the number of females, 3. the tenure length of the alpha male. Predictors for low rank achievers were: 1. Number of females in a group, 2. the tenure length of the alpha male. Since in both models two variables were compared with each other, binomial models were conducted using R (2.15.2)^49^. All data were z-transformed. We checked for influential cases using dfbeta. A value below 2 indicated the absence of influential cases in our data. We checked for co-linearity using variance inflation factors^50^ in the package car^51^ applied to a standard linear modal. With all VIFs being smaller than 2.2 in our models, collinearity between the predictors was not a problem^52^,^53^. Due to the limited sample size we additionally tested the data set with non-parametric tests (Mann–Whitney U-test). We controlled for multiple testing using the Benjamini–Hochberg correction^54^.

We used a Chi-square test to determine whether the timing of immigration in relation to other immigrations differed between males of high and low rank achievers. Differences between males of high and low rank achievers regarding their chance of getting injured were analysed using a Fisher exact test. All significance levels were set to two-tailed p-values < 0.05. All test were conducted in R^49^.

## Additional Information

**How to cite this article**: Marty, P. R. *et al.* Determinants of immigration strategies in male crested macaques (*Macaca nigra*). *Sci. Rep.* **6**, 32028; doi: 10.1038/srep32028 (2016).

## Figures and Tables

**Table 1:** Results of the GLM for high rank achievers in which the number of males as well as the tenure of the alpha male in the target groups was compared to that in potential other target groups.

Model 1	Estimate	SE	Z	P
Intercept	0.08	0.48	0.17	0.864
Number of males	1.80	0.70	2.57	0.010*
Number of females	−0.67	0.55	−1.26	0.221
Tenure of alpha male in the group	−0.19	0.50	−0.38	0.708

**Table 2:** Results of the GLM for low rank achievers in which the number of females as well as the tenure of the alpha male of the target groups was compared with that of potential other target groups.

Model 2	Estimate	SE	Z	P
Intercept	−2.58	1.57	−1.64	0.101
Number of females	−1.11	0.75	−1.48	0.140
Tenure of alpha male in the group	−9.72	4.73	−2.05	0.040*

**Table 3:** Number and composition of groups studied at the Tangkoko-Butuangus Nature Reserve.

Group	Number of adult females	Number of adult males	Number of immigrations	Number of emigrations	Study period	Follow days
R1	18–28	9–18	28	29	3.2006–12.2012	1854
R2	13–21	4–11	7	6	3.2006–12.2012	1452
R3	7	4	3	0	7.2012–12.2012	47
PB	15–25	7–10	19	18	8.2007–12.2012	1100

## References

[b1] Pusey A. E. Sex-biased dispersal and inbreeding avoidance in birds and mammals. Trends Ecol. Evol. 2, 295–299 (1987). 21227869 10.1016/0169-5347(87)90081-4

[b2] Henzi S. P. & Lucas J. W. Observations on the Inter-Troop Movement of Adult Vervet Monkeys (*Cercopithecus aethiops*). Folia Primatol. 33, 220–235 (1980). 7191819 10.1159/000155936

[b3] Clutton-Brock T. & Lukas D. The evolution of social philopatry and dispersal in female mammals. Mol. Ecol. 472–492 (2012). 21883582 10.1111/j.1365-294X.2011.05232.x

[b4] Alberts S. C. & Altmann J. Balancing costs and opportunities: dispersal in male baboons. Am. Nat. 145, 279–306 (1995).

[b5] Yoder J. M. The cost of dispersal: predation as a function of movement and site familiarity in ruffed grouse. Behav. Ecol. 15, 469–476 (2004).

[b6] Bonte D. *et al.* Costs of dispersal. Biol. Rev. Camb. Philos. Soc. 87, 290–312 (2012). 21929715 10.1111/j.1469-185X.2011.00201.x

[b7] Eraud C., Jacquet A. & Legagneux P. Post-Fledging Movements, Home Range, and Survival of Juvenile Eurasian Collared-Doves in Western France. Condor 113, 150–158 (2011).

[b8] Wiens J. D., Noon B. R. & Reynolds R. T. Post-Fledging Survival Of Northern Goshawks: The Importance Of Prey Abundance, Weather, And Dispersal. Ecol. Appl. 16, 406–418 (2006). 16705989 10.1890/04-1915

[b9] Teichroeb J. A., Wikberg E. C. & Sicotte P. Dispersal in male ursine colobus monkeys (*Colobus vellerosus*): influence of age, rank and contact with other groups on dispersal decisions. Behaviour 148, 765–793 (2011).

[b10] Cheney D. L. & Seyfarth R. M. Nonrandom Dispersal in Free-Ranging Vervet Monkeys: Social and Genetic Consequences. Am. Nat. 122, 392 (1983).

[b11] Zhao Q.-K. Etho-ecology of Tibetan macaques at Mount Emei, China in Evolution and Ecology of Macaques Societies (eds Fa J. E. & Lindburg D. G.) 263–289 (Cambridge University Press, 1996).

[b12] Pärt T. The importance of local familiarity and search costs for age- and sex-biased philopatry in the collared flycatcher. Anim. Behav. 49, 1029–1038 (1995).

[b13] Packer C. & Pusey A. E. Male takeovers and female reproductive parameters: A simulation of oestrous synchrony in lions (*Panthera leo*). Anim. Behav. 31, 334–340 (1983).

[b14] Gross M. R. Alternative reproductive strategies and tactics: diversity within sexes. Trends Ecol. Evol. 11, 92–98 (1996). 21237769 10.1016/0169-5347(96)81050-0

[b15] Brockmann H. J. The evolution of alternative strategies and tactics. Adv. Study Behav. 30, 1–51 (2001).

[b16] Pusey A. & Packer C. In Primate Sociesties (eds Smuts B. B., Cheney D. L., Seyfarth R. M., Wrangram R. W. & Struhsaker T. T.) 250–266 (Chicago University Press, 1987).

[b17] Smale L., Nunes S. & Holekamp K. Sexually dimorphic dispersal in mammals: patterns, causes, and consequences. Adv. Study Behav. 26, 181–250 (1997).

[b18] Jack K. Secondary Dispersal by Male Primates. Primate Rep. 67, 61–83 (2003).

[b19] van Noordwijk M. A. & van Schaik C. P. Male migration and rank acquisition in wild long-tailed macaques (*Macaca fascicularis*). Anim. Behav. 33, 849–861 (1985).

[b20] Melnick D. J., Pearl M. C. & Richard A. F. Male migration and inbreeding avoidance in wild rhesus monkeys. Am. J. Primatol. 7, 229–243 (1984). 10.1002/ajp.1350070303 32111112

[b21] Henzi S. P. & Lucas J. W. Observations on the inter-troop movement of adult vervet monkeys (*Cercopithecus aethiops*). Folia Primatol. 33, 220–235 (1980). 7191819 10.1159/000155936

[b22] van Noordwijk M. A. & van Schaik C. P. Career moves: transfer and rank challenge decisions by male long-tailed macaques. Behaviour 138, 359–395 (2001).

[b23] Zhao Q.-K. Mating competition and intergroup transfer of males in Tibetan macaques (*Macaca thibetana*) at Mt. Emei, China. Primates 35, 57–68 (1994).

[b24] Pope T. R. The evolution of male philopatry in neotropical monkeys in Primate Males: Causes and Consequences of Variation in Group Composition (ed. Kappeler P. M.) 219–235 (Cambridge university press, 2000).

[b25] Meikle D. B. & Vessey S. H. Nepotism among rhesus monkey brothers. Nature 294, 160–161 (1981). 7197754 10.1038/294160a0

[b26] Schoof V., Isbell L. & Jack K. What traits promote male parallel dispersal in primates? Behaviour 146, 701–726 (2009).

[b27] Cheney D. L. & Seyfarth R. M. How Monkeys See the World. (University of Chicago Press, 1990).

[b28] Jack K. M., Sheller C. & Fedigan L. M. Social factors influencing natal dispersal in male white-faced capuchins (*Cebus capucinus*). Am. J. Primatol. 7, 1–7 (2011). 10.1002/ajp.20974 21732399

[b29] Bergman T., Beehner J., Cheney D., Seyfarth R. & Whitten P. Correlates of stress in free-ranging male chacma baboons, Papio hamadryas ursinus. Anim. Behav. 70, 703–713 (2005).

[b30] Setchell J. M., Smith T., Wickings E. J. & Knapp L. A. Stress, social behaviour, and secondary sexual traits in a male primate. Horm. Behav. 58, 720–728 (2010). 20688067 10.1016/j.yhbeh.2010.07.004

[b31] Neumann C. *et al.* Assessing dominance hierarchies: validation and advantages of progressive evaluation with Elo-rating. Anim. Behav. 82, 911–921 (2011).

[b32] Schuster R., Raleigh M. J., McGuire M. T. & Torigoe D. Rank, relationships, and responses to intruders among adult male vervet monkeys. Am. J. Primatol. 31, 111–127 (1993). 10.1002/ajp.1350310204 31937002

[b33] van Noordwijk M. A. & van Schaik C. P. Sexual selection and the careers of primate males: paternity concentrations, dominance-acquisition tactics and transfer decisions in Sexual Selection in Primates (eds Kappeler P. M. & van Schaik C. P.) 208–229 (Cambridge university press, 2004).

[b34] Nakamichi M., Kojima Y., Itoigawa N., Imakawa S. & Machida S. Interactions among adult males and females before and after the death of the alpha male in a free-ranging troop of Japanese macaques. Primates 36, 385–396 (1995).

[b35] Borries C. Male dispersal and mating season influxes in Hanuman langurs living in multi-male groups in Primate Males: Causes and Consequences of Variation in Group Composition (ed. Kappeler P. M.) 146–158 (Cambridge university press, 2000).

[b36] Clarke P. M. R., Henzi S. P., Barrett L. & Rendall D. On the road again: competitive effects and condition-dependent dispersal in male baboons. Anim. Behav. 76, 55–63 (2008).

[b37] Yao H. *et al.* Male dispersal in a provisioned multilevel group of *Rhinopithecus roxellana* in Shennongjia Nature Reserve, China. Am. J. Primatol. 73, 1280–1288 (2011). 21898518 10.1002/ajp.21000

[b38] Zhao Q., Borries C. & Pan W. Male takeover, infanticide, and female countertactics in white-headed leaf monkeys (*Trachypithecus leucocephalus*). Behav. Ecol. Sociobiol. (2011).

[b39] Marty P. R., Hodges K., Agil M. & Engelhardt A. Alpha male replacements and delayed dispersal in crested macaques (*Macaca nigra*). Am. J. Primatol. doi: 10.1002/ajp.22448 (In press). PMC5484350 26194621

[b40] van Schaik C. P., van Noordwijk M. & Nunn C. Sex and social evolution in primates in Comparative primate socioecology (ed. Lee P.) 204–231 (Cambridge university press, 1999).

[b41] Hamilton W. & Bulger J. Natal male baboon rank rises and successful challenges to resident alpha males. Behav. Ecol. Sociobiol. 26, 357–362 (1990).

[b42] Muroyama Y., Imae H. & Okuda K. Radio tracking of a male Japanese macaque emigrated from its group. Primates 41, 351–356 (2000). 10.1007/BF02557604 30545186

[b43] Gerber L., Krützen M., de Ruiter J. R., van Schaik C. P. & van Noordwijk M. A. Postdispersal nepotism in male long-tailed macaques (*Macaca fascicularis*). Ecol. Evol. 6, 46–55 (2016). 26811773 10.1002/ece3.1839 PMC4716510

[b44] Rosenbaum B., O’Brien T. G., Kinnaird M. & Supriatna J. Population densities of Sulawesi crested black macaques (*Macaca nigra*) on Bacan and Sulawesi, Indonesia: effects of habitat disturbance and hunting. Am. J. Primatol. 44, 89–106 (1998). 9503122 10.1002/(SICI)1098-2345(1998)44:2<89::AID-AJP1>3.0.CO;2-S

[b45] Taylor W. Change-point analysis: A powerful new tool for detecting changes. Available at: www.variation.com/cpa/tech/changepoint.html. (Accessed August 26, 2014).

[b46] Altmann J. Observational study of behavior: sampling methods. Behaviour 49, 227–267 (1974). 4597405 10.1163/156853974x00534

[b47] de Vries H., Stevens J. M. G. & Vervaecke H. Measuring and testing the steepness of dominance hierarchies. Anim. Behav. 71, 585–592 (2006).

[b48] Leiva D. & de Vries H. Testing Steepness of Dominance Hierarchies. (R Package) (2011).

[b49] R Development Core Team. R: A language and environment for statistical computing (2009).

[b50] Field A. Discovering statistics using SPSS. (Sage Publications 2005).

[b51] Fox J. & Weisberg H. S. An R companion to applied regression. (Saga Publications 2010).

[b52] Bowerman B. & O’Connell R. Linear statistical models: An applied approach. (Duxbury 1990).

[b53] Myers R. H. Classical and modern regression with applications. (Duxbury 1990).

[b54] Benjamini Y. & Hochberg Y. Controlling the False Discovery Rate : A Practical and Powerful Approach to Multiple Testing. R. Stat. Soc. 57, 289–300 (1995).

